# Developmental Patterning and Neurogenetic Gradients of *Nurr1* Positive Neurons in the Rat Claustrum and Lateral Cortex

**DOI:** 10.3389/fnana.2021.786329

**Published:** 2021-12-02

**Authors:** Chao Fang, Hong Wang, Robert Konrad Naumann

**Affiliations:** CAS Key Laboratory of Brain Connectome and Manipulation, The Brain Cognition and Brain Disease Institute (BCBDI), Shenzhen Institute of Advanced Technology, Chinese Academy of Sciences, Shenzhen-Hong Kong Institute of Brain Science-Shenzhen Fundamental Research Institutions, Shenzhen, China

**Keywords:** claustrum, EdU, birth dating, rat, *Nurr1*, *Nr4a2*, cortical development

## Abstract

The claustrum is an enigmatic brain structure thought to be important for conscious sensations. Recent studies have focused on gene expression patterns, connectivity, and function of the claustrum, but relatively little is known about its development. Interestingly, claustrum-enriched genes, including the previously identified marker *Nurr1*, are not only expressed in the classical claustrum complex, but also embedded within lateral neocortical regions in rodents. Recent studies suggest that *Nurr1* positive neurons in the lateral cortex share a highly conserved genetic expression pattern with claustrum neurons. Thus, we focus on the developmental progression and birth dating pattern of the claustrum and *Nurr1* positive neurons in the lateral cortex. We comprehensively investigate the expression of *Nurr1* at various stages of development in the rat and find that *Nurr1* expression first appears as an elongated line along the anterior-posterior axis on embryonic day 13.5 (E13.5) and then gradually differentiates into multiple sub-regions during prenatal development. Previous birth dating studies of the claustrum have led to conflicting results, therefore, we combine 5-ethynyl-2′-deoxyuridine (EdU) labeling with *in situ* hybridization for *Nurr1* to study birth dating patterns. We find that most dorsal endopiriform (DEn) neurons are born on E13.5 to E14.5. Ventral claustrum (vCL) and dorsal claustrum (dCL) are mainly born on E14.5 to E15.5. *Nurr1* positive cortical deep layer neurons (dLn) and superficial layer neurons (sLn) are mainly born on E14.5 to E15.5 and E15.5 to E17.5, respectively. Finally, we identify ventral to dorsal and posterior to anterior neurogenetic gradients within vCL and DEn. Thus, our findings suggest that claustrum and *Nurr1* positive neurons in the lateral cortex are born sequentially over several days of embryonic development and contribute toward charting the complex developmental pattern of the claustrum in rodents.

## Introduction

The claustrum is an irregularly shaped sheet of neurons located in the basolateral forebrain of the mammalian brain. It is thought to be involved in four main functions: regulation of sleep, consciousness, attention and salience, and memory (Mathur, 2014; Jackson et al., 2020; Smith et al., 2020; Wong et al., 2021). This notion is supported by the dense connectivity of the claustrum with other cortical regions (Smith and Alloway, 2010; Patzke et al., 2014; Torgerson et al., 2015; Kitanishi and Matsuo, 2017; Wang et al., 2017; Zingg et al., 2018). In addition, the claustrum also shows a wide range of shapes among mammals (Johnson et al., 2014; Binks et al., 2019), which has made it a challenge to define its precise anatomical boundaries, especially in rodents (Dillingham et al., 2019; Smith et al., 2019). But what exactly is the claustrum? In several anatomical studies (e.g., Witter et al., 1988), the claustrum is relatively broadly defined, encompassing a range of nuclei that are now identified as distinct subregions such as the dorsal claustrum (dCL), ventral claustrum (vCL), and the dorsal endopiriform nucleus (DEn). Much like other cortical regions, the claustrum consists of roughly 90% glutamatergic neurons (Druga, 2014), and typical claustrum markers like *Nurr1* (also known as *Nr4a2*) label a large fraction of these neurons (Erwin et al., 2021). Recent studies of the claustrum using circuit-based and transcriptomic approaches (Wang et al., 2017; Erwin et al., 2021; Marriott et al., 2021; Peng et al., 2021) allow a more precise definition of the claustrum and related nuclei. Intriguingly, while there is an ever expanding list of gene markers mainly expressed in the claustrum (Zeisel et al., 2015; Wang et al., 2017; Smith et al., 2019; Bruguier et al., 2020; Erwin et al., 2021; Yao et al., 2021), a large fraction of these genes are expressed not only in the claustrum proper, but also consistently in other regions, such as the DEn, the subplate (Sp), and – in rodents – in the deep layers of lateral neocortex (Arimatsu, 1994; Puelles, 2014; Bruguier et al., 2020; Ratié et al., 2020). Knowledge about the developmental patterning of claustrum cell types could help to understand their adult distribution and function. However, despite the wealth of new gene expression data, the development of claustrum cell types remains incompletely understood.

In mammals, the cerebral cortex and several non-layered regions that largely consist of excitatory neurons together comprise the pallium, or mantle, of the telencephalon (Puelles et al., 2013). The pallium is best understood as a system of concentric rings of variable differentiation in terms of layers, connections, and cell types, with the neocortex at the center (Filimonoff, 1947; Sanides, 1970; Puelles et al., 2019; Medina et al., 2021). In this model, surrounding the core neocortical sensory and motor areas are transition regions, such as, the insular, perirhinal, or cingulate cortices. These regions show less differentiated lamination, closer interconnections with the amygdala or hippocampal regions, a distinct temporal pattern of development, and a distinct roster of cell types compared to the core neocortex (Reep, 1984; Bayer, 1990; Yao et al., 2021). The lateral transition areas and ventral to it, the olfactory cortex, have a unique characteristic among cortical regions: a set of embedded nuclei – the claustrum and the endopiriform nucleus – consistently located at the same topological position across all mammals (Binks et al., 2019). Given the distinct gene expression patterns in the claustrum and endopiriform nucleus compared to their surrounding cortical areas, elucidating their developmental origin can help to understand structure, connectivity, and possibly function in the adult brain. Puelles et al. (1999, 2000) proposed a model of the pallium containing four subdivisions or sectors, each of which is generated from progenitor cells at different positions of the developing forebrain. Recently, *Nurr1* expression was instrumental in updating the pallial sector model (Puelles, 2014, 2017; Puelles et al., 2016a; Watson and Puelles, 2017). Its current version stipulates that *Nurr1* positive neurons in the claustrum belong to the lateral sector of the pallium, together with neurons from the insular cortex. Other cortical *Nurr1* positive neurons outside the insular cortex are also proposed to be closely related to claustrum neurons, arriving at their final position by tangential migration in the ventral (DEn) or dorsal (dCL, deep layers of lateral neocortex) direction. Thus, unlike most other pallial neurons which only migrate radially, claustrum and endopiriform nucleus neurons may have both unique gene expression and migratory patterns.

A key aspect of claustrum development is to understand the temporal sequence of claustrum neuron generation. Several authors studied birth dating of claustrum and endopiriform nucleus neurons using injections of [H^3^]thymidine or 5-bromo-2′-deoxyuridine (BrdU) in the rat (reviewed in Puelles, 2014; Bruguier et al., 2020). Interestingly, a wide spectrum of birth dates was reported, with some studies observing early claustrum neuron production peaking at embryonic day 11.5 (E11.5) or E12.5 (Bisconte and Marty, 1975; Valverde and Santacana, 1994) and others observing peak production at E14–E16 (Bayer and Altman, 1991; Arimatsu, 1994; Arimatsu et al., 2003). Bayer and Altman (1991) investigated the patterning of neurogenesis with the aim to identify gradients of neuron generation within the claustrum and DEn. They showed that dorsal endopiriform neurons develop between E12 and E17, with peak production in ventral parts at E14 and in more dorsal parts at E15. In contrast, the claustrum displays a posterior to anterior gradient. In the posterior claustrum, most neurons are born on E15, while anteriorly more neurons are generated at E16. The first studies to combine birth dating with claustrum-specific markers (Arimatsu, 1994; Arimatsu et al., 2003) identified comparable birth dates for the dorsal endopiriform nucleus and the ventral claustrum but did not discuss neurogenetic gradients within these regions. Instead, Arimatsu (1994) and Arimatsu et al. (2003) focused on deep layer neurons (dLn) of the lateral neocortex expressing the claustrum marker Latexin (Lxn), and found that most of these neurons are born on E15, unlike other deep layer neocortex neurons. To our knowledge, the birth dates of the dorsal claustrum neurons have not been studied. Some of the variability in birth dates may be attributable to differences in defining embryonic dates or injection patterns of thymidine analogs (see Puelles, 2014), some to definitions of claustrum and dorsal endopiriform sub-regions, and some to methodological differences. For example, both [H^3^]thymidine and BrdU labeling involve tissue processing steps that may impact co-labeling with antibodies or *in situ* hybridization (Miller and Nowakowski, 1988). More recent birth dating methods, such as 5-ethynyl-2′-deoxyuridine (EdU) and FlashTag labeling (Salic and Mitchison, 2008; Govindan et al., 2018) or neurogenetic tagging techniques based on Cre-loxP recombination (Hirata et al., 2019, 2021) have the potential to clarify claustrum birth dating but have not been used to that end. In conclusion, previous studies identified a wide range of claustrum birth dates while considering only some of the regions now known to express claustrum specific markers.

Recently, Hedderich et al. (2021) observed altered claustrum microstructure in the human brain after premature birth. The authors suggest that perinatal development influences the cellular architecture of the claustrum which in turn could be relevant for explaining impaired cognitive performance in premature-born adults. These findings highlight the need to further our understanding about normal and aberrant claustrum development. Therefore, the aims of the present study are: (i) to determine the distribution of *Nurr1* expressing neurons in the prenatal and early postnatal rat cerebral cortex, (ii) to identify stable and transient patterns of *Nurr1* expression, and (iii) to establish a comprehensive birth dating profile of *Nurr1* expressing claustrum and *Nurr1* positive neurons in the lateral cortex. To accomplish these aims, we combine single EdU injections on consecutive days during embryonic development with *in situ* hybridization detection of *Nurr1* throughout the entire developing rat brain. Most previous studies of claustrum birth dating have focused on the rat as a model animal, yet shown widely diverging results. To clarify and compare results, here, we also focus on the rat. In addition, we study EdU signals at birth, because all claustrum subdivisions and related cell types are readily identifiable, including some which only appear transiently.

## Materials and Methods

### Animals

Experimental procedures were performed in accordance with the guidelines of the Animal Care and Use Committees at the Shenzhen Institute of Advanced Technology (SIAT), Chinese Academy of Sciences (CAS), China (permit number SIAT-IACUC-20210607-NS-NBJZX-ROBERT NAUMANN-A1706-01). Wild type pregnant Sprague Dawley (SD) rats were supplied by Beijing Vital River Laboratory Animal Technology Co., Ltd. Both male and female rats aged from E13.5 to postnatal day 20 (P20) were used in this study. Food and water were given *ad libitum*. The day females were sperm positive was designated as E0.5. Pups were typically born on E21.5-E22.5, and the birth day was designated as P0.

### EdU Injection

5-ethynyl-2′-deoxyuridine (EdU, ApexBIO, Houston, United States, Cat# B8337) was diluted in 0.1 M phosphate buffered saline solution (PBS). Female timed-pregnant rats (*N* = 18, 3 per group, bodyweight 280–320 g) received a single 25 mg/kg EdU injection given between 17:00 and 18:00 on days corresponding to E12.5 to E17.5. At birth, all newborn rats were perfused.

### Tissue Preparation

Pregnant rats or rat pups (P0, P2, P4, P8 and P20) were briefly anesthetized with isoflurane and subsequently given an intraperitoneal injection of 60 mg/kg pentobarbital. Rat pups were perfused with 0.1 M DEPC (diethyl pyrocarbonate)-PBS and 4% paraformaldehyde (PFA) in 0.1 M phosphate buffer (PB), followed by brain dissection and fixation with 4% PFA overnight. Embryonic brains were dissected out in ice cold DEPC-PBS, followed by fixation in 4% PFA overnight. Before sectioning, rat brains were cryoprotected using a 30% sucrose solution in DEPC-PBS for over 24 h. We used OCT compound for embedding brains and prepared 50 μm thick coronal sections.

### *In situ* Hybridization

*In situ* hybridization was performed using a protocol based on Ferran et al. (2015). DIG-labeled Riboprobes were used for hybridization on 50 μm free floating cryosections. Hybridization was performed overnight at 65°C without proteinase K treatment. Sections were washed at 65°C two times in 2xSSC/50% Formamide/0.1% N-lauroylsarcosine for 20 min, twice in 2xSSC/0.1% N-lauroylsarcosine at 37°C for 20 min and twice in 0.2xSSC/0.1% *N*-lauroylsarcosine at 37°C for 20 min. Sections were blocked in MABT/10% goat serum/1% Blocking reagent (Roche Diagnostics, Mannheim, Germany, Cat# 11096176001), incubated overnight with sheep anti-DIG-AP (1:1000, Roche Diagnostics, Mannheim, Germany, Cat# 11093274910). After washing, staining was performed using NBT/BCIP in NTMT until satisfactory intensity was reached. Staining reaction was stopped with 10 mM EDTA. Sections were washed, dehydrated and mounted with Eukitt^®^ Quick-hardening mounting medium (Sigma Aldrich, St. Louis, United States, Cat# 03989).

Fluorescent *in situ* hybridization was performed using DIG-labeled probes. After hybridization and washing, sections were first incubated with sheep anti-DIG-POD (1:1000, Roche Diagnostics, Mannheim, Germany, Cat# 11207733910) and tyramide signal amplification (TSA) was performed using biotin-tyramide (ApexBIO, Houston, United States, Cat#A8011). For TSA, sections were equilibrated 5 min in TSA buffer (10 mM imidazole in PBS), then incubated in the TSA staining solution (2 μg/ml biotin-tyramid/0.001%H_2_O_2_/TSA buffer) in the dark for 30 min without shaking. After washing, sections, subsequently, were incubated with Streptavidin-Cy2 (Jackson ImmunoResearch) to detect the DIG labeled probe. Sections were washed, then mounted with Fluromount medium (Sigma Aldrich, St. Louis, United States, Cat# F4680).

Rat cDNA was synthetized from total brain RNA using EasyScript First-Strand cDNA Synthesis SuperMix (Transgen Biotech, Beijing, China, Cat# AE301-02). The *Nurr1* probe was amplified with the following primer pairs: Fw-tgttcaggcgcagtatgggtcc. Re-tcacaagtgcgaacaccgtaatgc, for a total length of 834 bp, and then cloned in a pEASY-Blunt Zero backbone (Transgen Biotech, Beijing, China, Cat# CB501-01) and verified by sequencing. Antisense digoxigenin-labeled riboprobes were synthetized according to the protocol recommended by the manufacturer (Roche Diagnostics, Mannheim, Germany).

### EdU Click Chemistry Reaction

Detection of EdU was performed after *in situ* hybridization with a modified protocol based on Salic and Mitchison, 2008 and Hong et al., 2009. Free-floating sections were washed 3 × 5 min in PBS, 1 × 20 min 0.5% Triton X-100/PBS, 3 × 5 min in PBS. Subsequently, sections were incubated for 30 min in the EdU click chemistry reaction buffer in the dark at room temperature. The reaction buffer (working solution) was prepared freshly by combining in the following order: (1) 0.1 M PBS (2) 5 mM CuSO4, (3) 1 mM THPTA (tris-hydroxypropyltriazolylmethylamine), (4) 30 mM Na-Asc (Ascorbic Acid Sodium Salt), and (5) 25 μM Cy3 azide (ApexBIO, Cat# A8127). When preparing the reaction buffer, mix well after adding each component, the solution should be transparent after adding Na-Asc. After the click reaction, sections were washed 3 × 5 min in PBS. A detailed ISH and EdU labeling protocol can be found online https://wangnaumannlab.cn/wj.

### Quantification of *Nurr1* + EdU + Double Positive Cells

For quantification of cells expressing both *Nurr1* and EdU, we use P0 sections where all *Nurr1* labeled sub-regions can be clearly identified and are comparable across animals. For each individual region, we counted double positive cells from three animals in three equally distanced sections at the coordinates indicated below. For the ventral DEn (vDEn), dorsal DEn (dDEn), vCL, and dCL, we use anterior sections at bregma 0.8 to 0.6 mm. For the dLn, we use intermediate sections at bregma −0.4 to −0.6 mm. For the superficial layer neurons (sLn) and posterior DEn (pDEn), we use posterior sections at bregma −1.6 to −1.8 mm (coordinates based on Khazipov et al., 2015). We focus on injection dates with significant EdU expression for each subregion: E12.5 to E15.5 for the vDEn, dDEn and pDEn; E13.5 to E15.5 for the vCL and dCL; E13.5 to E16.5 for the dLn and E15.5 to E17.5 for the sLn.

### Image Acquisition

All chromogenic and fluorescent stainings were imaged at 10 × or 20 × magnification on a slide scanner (BX61VS, Olympus, Japan) or at 20 × magnification on an ApoTome microscope (Axio Imager 2, ZEISS, Germany). The fluorescent images were acquired in monochrome, and color maps were applied to the images post acquisition. *Post hoc* linear brightness and contrast adjustment were applied uniformly to the image under analysis.

## Results

### EdU Labeling of Dividing Neurons and *Nurr1* Labeling of Claustrum Related Regions

**FIGURE 1:**
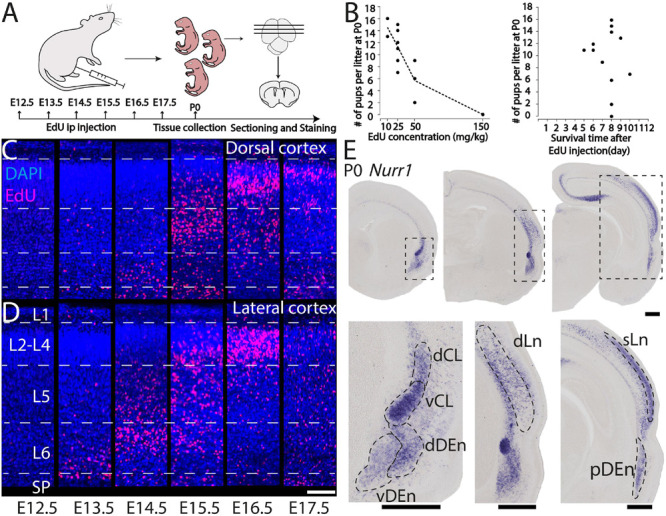
Overview of technical approach. **(A)** Schematic diagram of EdU injections in pregnant rats from E12.5 to E17.5, followed at P0 by tissue collection, microtomy, and staining. **(B)** Relation of P0 rats survival rate with EdU concentrations and (left graph), and with survival time after EdU injection (right graph). EdU labeled P0 dorsal **(C)** and lateral **(D)** cortical sections from E12.5 to E17.5 injections. **(E)** *Nurr1* expression in P0 sections from anterior to posterior, and magnified *Nurr1* labeled regions including, dCL (dorsal claustrum), vCL (ventral claustrum), dDEn (dorsal DEn), vDEn (ventral DEn), dLn (deep layer neurons), sLn (superficial layer neurons), and pDEn (posterior DEn). Scale bar in panels **(C,D)** = 100 μm. Scale bar in panel **(E)** = 0.5 mm.

We study the birth dating of the claustrum, endopiriform nucleus, and related cortical regions that are labeled by *Nurr1* gene expression by injecting EdU at different time points in timed-pregnant rats. We define E0.5 as the first day that females were sperm positive. We investigate the distribution of EdU labeling and *Nurr1* expression in rat pups at birth P0 (Figure 1A). Since EdU toxicity may adversely affect cellular health (Andersen et al., 2013), we sought to identify the lowest dose of EdU that allows reliable birth dating while maximizing pup survival. We find that injections of 10 mg/kg or 25 mg/kg EdU show little effects on litter size, while higher doses correlate with reduced litter size (Figure 1B). The survival time after EdU injection was not correlated with litter size (Figure 1B). Thus, we subsequently use injections of 25 mg/kg for all further experiments. Figures 1C,D shows the lateral and dorsal regions of cortex of P0 rats after consecutively timed EdU injections. In the dorsal cortex (Figure 1C), we observe EdU signal in cortical layer 6 mainly in animals injected on E14.5, in layer 5 mainly in animals injected on E15.5 and in upper layers in animals injected on E16.5 and E17.5. In contrast, in the lateral cortex (Figure 1D) EdU signals appeared earlier and relatively few newborn neurons were detected in animals injected later than E16.5. To identify claustrum neurons we use *in situ* hybridization for *Nurr1*, a relatively selective and early expressed marker (Puelles, 2014). We study seven brain regions labeled by *Nurr1* illustrated in three coronal sections of the rat brain at P0 (Figure 1E). In the anterior section, *Nurr1* labels four sub-regions, including dCL and vCL. Since the DEn shows dark and light staining parts, we divide it into dDEn, and vDEn as suggested previously (Bayer and Altman, 1991; Arimatsu, 1994). In the intermediate section, we identify *Nurr1* expression in cortical deep layers dLn. In the posterior section, *Nurr1* is expressed in cortical superficial layers sLn, and the elongated pDEn. Another region containing *Nurr1* positive neurons, the Sp has been studied in great detail (reviewed in Hoerder-Suabedissen and Molnár, 2015; Molnár et al., 2020), so we do not investigate it here.

### Prenatal *Nurr1* Expression in the Lateral Forebrain

**FIGURE 2:**
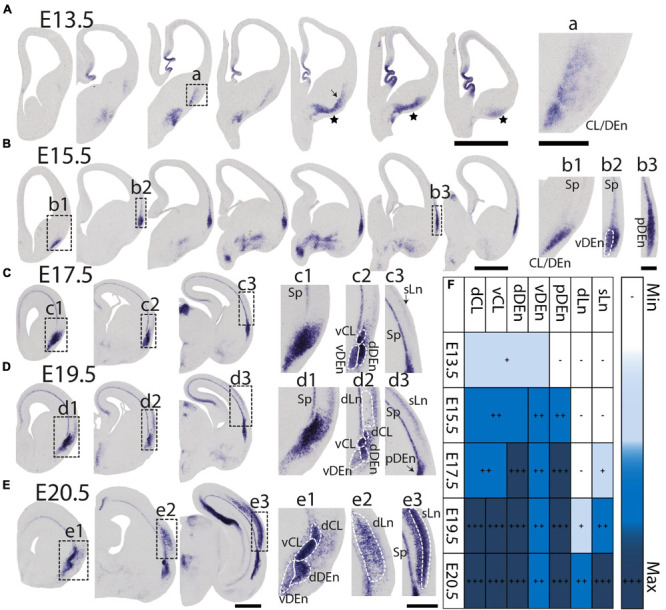
Prenatal development of *Nurr1* expression. *Nurr1* expression on E13.5 **(A)**, on E15.5 **(B)**, on E17.5 **(C)**, on E19.5 **(D)** and on E20.5 **(E)**. High magnification *Nurr1* labeled regions from E13.5 to E20.5 (a–e), including the CL/DEn on E13.5, the Sp, vDEn, and pDEn on E15.5, the sLn on E17.5, the dLn, vDEn, dDEn, vCL and dCL on E19.5, and all sub-regions on E20.5. **(F)** Table of prenatal development of *Nurr1* expression. White and “–” indicate no expression, light blue and “ + ” indicate beginning *Nurr1* expression, higher expression is shown in darker color and more “ + ” signs. *N* = 2 rats for each date. Scale bars in panels **(A–E)** = 1 mm. Scale bars in panels (a–b) = 200 μm. Scale bars in panels (c–e) = 500 μm.

During prenatal development, we observe the earliest *Nurr1* expression in the prospective claustrum and dorsal endopiriform regions (CL/DEn) on E13.5 (Figure 2A). In intermediate sections, *Nurr1* first appears as a small elongated region. Its ventral part is located closer - its dorsal part more distant to the brain surface (Figure 2Aa). In more posterior sections, we observe little *Nurr1* staining in the lateral forebrain yet a large group of *Nurr1* positive cells is present at more ventral locations marked with a star symbol. We observe a direct connection of this ventral posterior *Nurr1* positive region and the prospective CL/DEn region marked by an arrow (Figure 2A). E15.5 (Figure 2B) sees a major shift in the intensity and distribution *Nurr1* expression. *Nurr1* now labels the CL/DEn in more anterior and more posterior sections than in earlier stages, also weakly labeled in the lateral part of the cortex. Sp neurons are also weakly labeled. Anteriorly, CL/DEn has reversed its orientation. Its dorsal part is now closer to the brain surface while the ventral part is relatively distant from the brain surface (Figure 2Bb1). In the intermediate sections the CL/DEn starts to divide into two parts with different staining intensity (Figure 2Bb2). The elongated shape of the pDEn becomes apparent in more posterior sections (Figure 2Bb3). From E17.5 (Figure 2C) until birth the *Nurr1* expression pattern in the prospective CL/DEn shows less overall changes, but becomes more differentiated. Anteriorly, different subregions of CL/DEn are not yet apparent on E17.5 (Figure 2Cc1) but at more intermediate levels more subregions become apparent, we tentatively label them as vCL, dDEn, and vDEn (Figure 2Cc2). In posterior sections, the pDEn appears continuously with the Sp neurons (Figure 2Cc3), while at intermediate sections there is a small gap between CL and Sp neurons and in anterior sections, a wider gap (Figures 2Cc1,c2). In addition, *Nurr1* starts to weakly label the sLn (Figure 2Cc3). On E19.5 (Figure 2D), *Nurr1* staining becomes more differentiated, with the vCL and dCL subdivisions visible (Figure 2Dd2). The gap between the brain surface and the CL, DEn, and pDEn keeps widening. In intermediate sections, the dLn shows first staining, and in posterior sections the sLn shows stronger *Nurr1* expression (Figures 2Dd2,d3). On E20.5 (Figure 2E), all sub-regions that we defined based on *Nurr1* expression in P0 brains are readily apparent (Figures 2Ee1–e3). In summary (Figure 2F), on E13.5 *Nurr1* first shows weak expression in a relatively ventral position. A major change occurs from E13.5 to E15.5, when *Nurr1* shows stronger expression in an elongated stripe from anterior to posterior and subdivisions of different staining intensity become visible. In the sLn, *Nurr1* shows weak expressions on E17.5 and increased expression on E19.5. On E20.5, *Nurr1* strongly labels all claustrum sub-regions.

### Postnatal *Nurr1* Expression in the Lateral Forebrain

**FIGURE 3:**
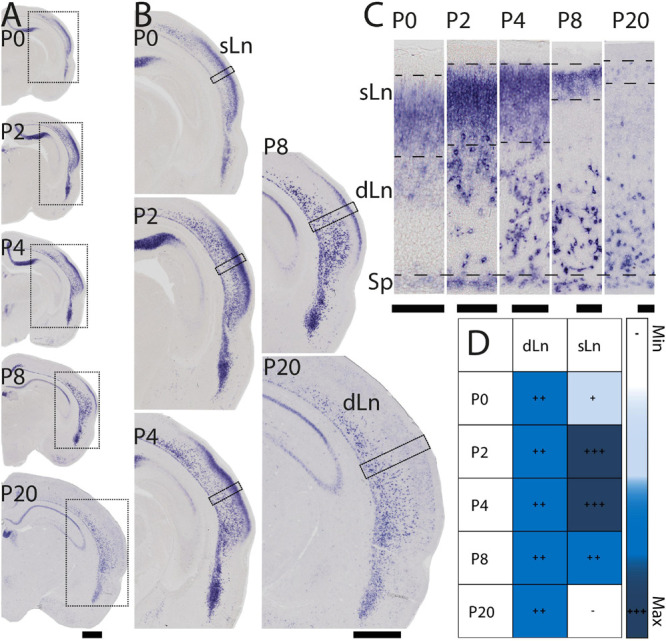
Early postnatal development of *Nurr1* expression in posterior cortical regions. **(A)** *Nurr1* expression on P0, P2, P4, P8, and P20. **(B)** Higher magnification *Nurr1* labeled regions from P0 to P20. **(C)** Higher magnification images of the columns indicated by black squares in panel **(B)** showing dLn and sLn from P0 to P20. **(D)** Table of early postnatal development of *Nurr1* expression in sLn. White and “–” indicate no expression, light blue and “ + ” indicate little expression, higher expression is shown in darker color and more “ + ” signs. *N* = 2 rats for each date. Scale bar in panels **(A,B)** = 1 mm. Scale bar in panel **(C)** = 100 μm.

During early postnatal development *Nurr1* expression in the claustrum and adjacent nuclei remains largely unchanged from its pattern at birth (Figure 1E). However, *Nurr1* expression in the posterior cortex changes dramatically during early postnatal development (Figure 3A). *Nurr1* expression in the sLn is greatly reduced from P2 to P20 (Figure 3B). On P2, *Nurr1* has the strongest expression in superficial layers, while on P20, there is almost no *Nurr1* expression in that region (Figure 3C). At birth, *Nurr1* expression in dLn neurons shows some distance from the Sp, while on P20, *Nurr1* expression in dLn is closer to the Sp (Figure 3C). We also note the approximately 3-fold expansion in cortical thickness taking place from P0 to P20 (see scale bars in Figure 3C). In summary, *Nurr1* expression in the sLn differs from other *Nurr1* positive populations we describe, in that its expression level is greatly reduced during early postnatal development (Figure 3D). Further, dLn neurons differ from other *Nurr1* positive neurons in changing their apparent laminar position during early postnatal development. Around birth, they are located mostly in the upper half of the cortex, intermingled with sLn cells, while on P20 they are located in the deep layers of lateral cortical regions. We did not observe major changes in *Nurr1* expression patterns after P20.

### Birth Dating of *Nurr1* Positive Cells in the Lateral Forebrain

**FIGURE 4:**
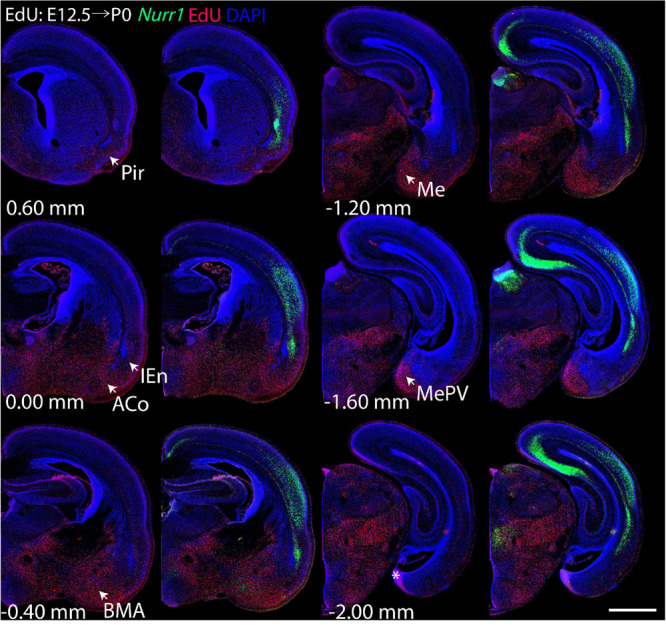
Overview of EdU labeling on E12.5 with *Nurr1* FISH on P0. White arrows indicate EdU labeled regions. Pir, piriform cortex, IEn, intermediate endopiriform nucleus, ACo, anterior cortical amygdaloid area, BMA, basomedial amygdaloid nucleus, anterior part, Me, medial amygdaloid nucleus, MePV, medial amygdaloid nucleus, posteroventral part. Numbers below sections indicate the bregma coordinate. *N* = 3 rats. “^∗^” indicates staining artifacts. Scale bar = 1 mm.

**FIGURE 5:**
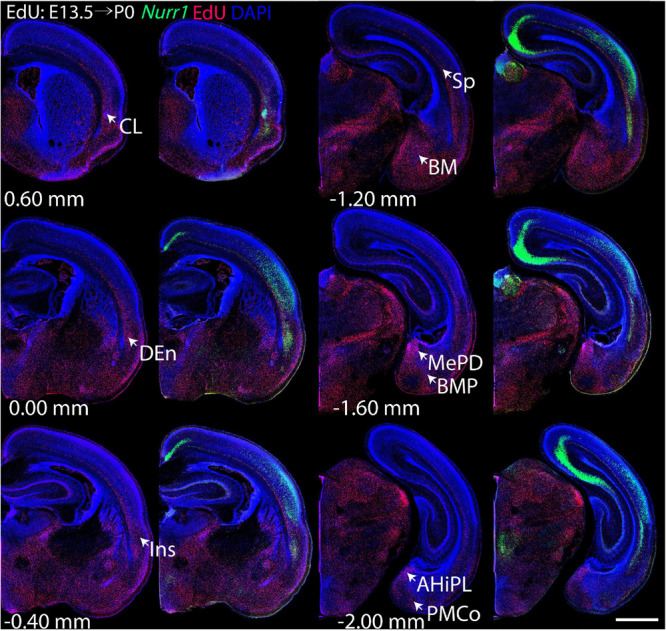
Overview of EdU labeling on E13.5 with *Nurr1* FISH on P0. White arrows indicate the EdU labeled regions. CL, claustrum, DEn, dorsal endopiriform nucleus, Ins, insular cortex, Sp, subplate, BM, basomedial amygdaloid nucleus, MePD, medial amygdaloid nucleus, posterodorsal part, BMP, basomedial amygdaloid nucleus, posterior part, AHiPL, amygdalohippocampal area, posterolateral, PMCo, posteromedial cortical amygdaloid area. Numbers below sections indicate the bregma coordinate. *N* = 3 rats. Scale bar = 1 mm.

**FIGURE 6:**
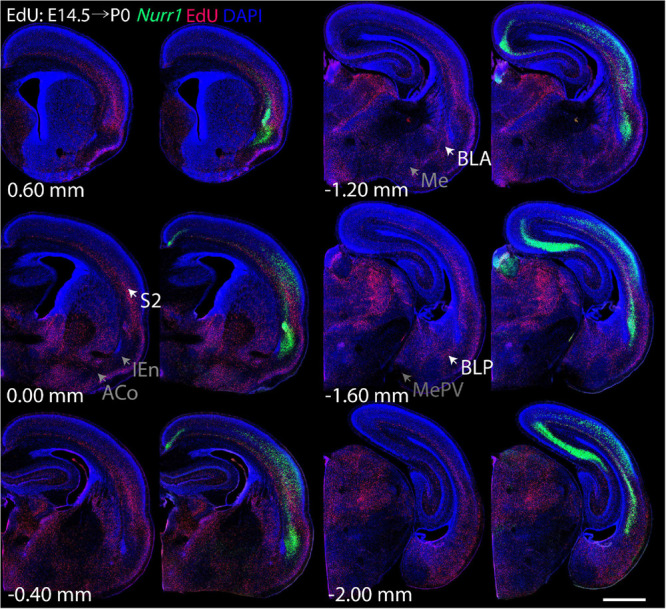
Overview of EdU labeling on E14.5 with *Nurr1* FISH on P0. White arrows indicate the EdU labeled regions. Gray arrows indicate the regions with reduced EdU labeling. S2, secondary somatosensory cortex, BLA basolateral amygdaloid nucleus, anterior part, BLP basolateral amygdaloid nucleus, posterior part, IEn, intermediate endopiriform nucleus, ACo anterior cortical amygdaloid area, Me, medial amygdaloid nucleus, MePV, medial amygdaloid nucleus, posteroventral part. Numbers below sections indicate the bregma coordinate. *N* = 3 rats. Scale bar = 1 mm.

**FIGURE 7:**
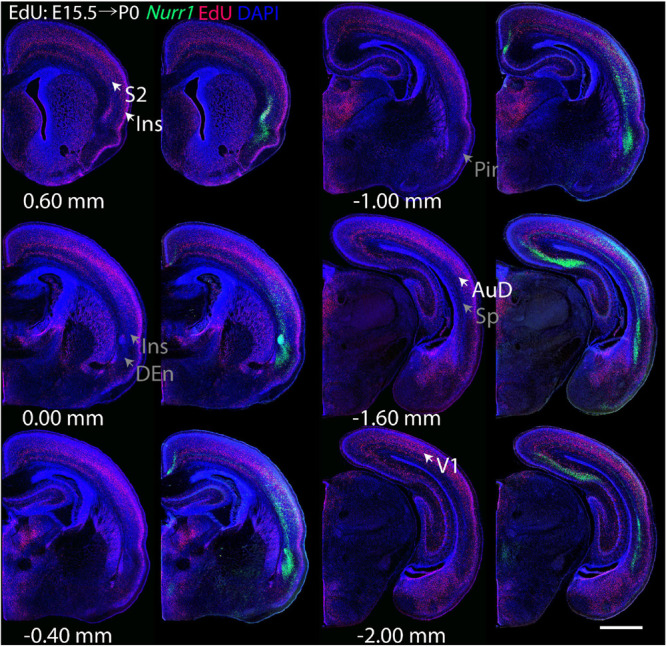
Overview of EdU labeling on E15.5 with *Nurr1* FISH on P0. White arrows indicate the EdU labeled regions. Gray arrows indicate the regions with reduced EdU labeling. S2, secondary somatosensory cortex, AuD secondary auditory cortex, V1 primary visual cortex dorsal area, DEn, dorsal endopiriform nucleus, Ins, insular cortex, Pir, piriform cortex, Sp, subplate. Numbers below sections indicate the bregma coordinate. *N* = 3 rats. Scale bar = 1 mm.

**FIGURE 8:**
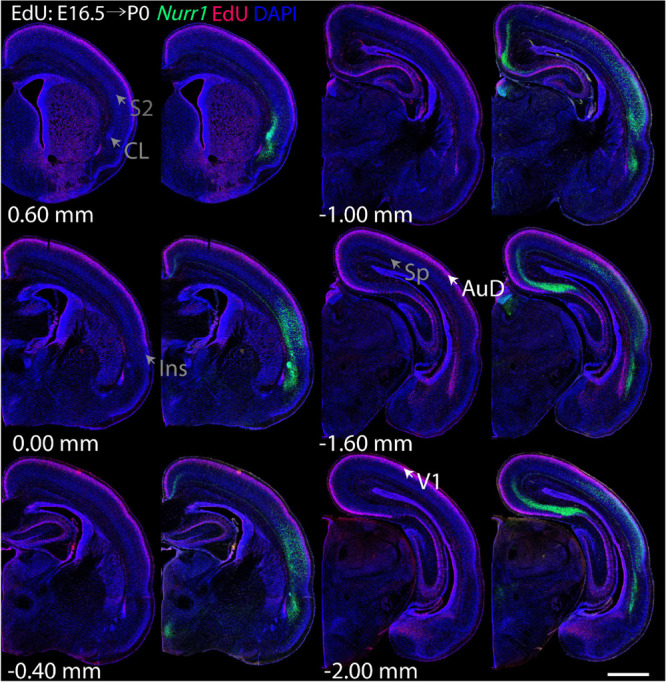
Overview of EdU labeling on E16.5 with *Nurr1* FISH on P0. White arrows indicate the EdU labeled regions. Gray arrows indicate the regions with reduced EdU labeling. AuD secondary auditory cortex, V1, primary visual cortex dorsal area, S2, secondary somatosensory cortex, CL, claustrum, Ins, insular cortex, Sp, subplate. Numbers below sections indicate the bregma coordinate. *N* = 3 rats. Scale bar = 1 mm.

**FIGURE 9:**
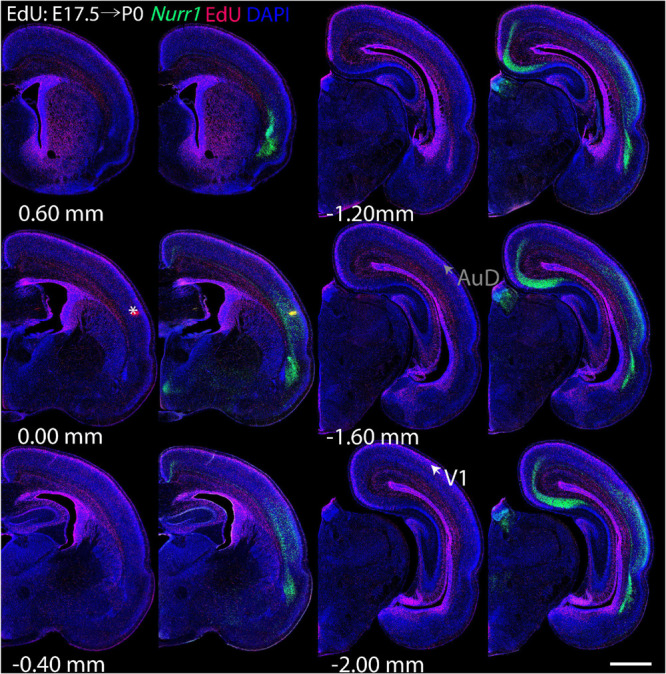
Overview of EdU labeling on E17.5 with *Nurr1* FISH on P0. White arrows indicate the EdU labeled regions. Gray arrows indicate the regions with reduced EdU labeling. AuD secondary auditory cortex, V1, primary visual cortex dorsal area. “^∗^” indicates staining artifacts. Numbers below sections indicate the bregma coordinate. *N* = 3 rats. Scale bar = 1 mm.

For each EdU injection date from E12.5 to E17.5, we selected 6 sections at comparable distances from anterior to posterior to show the EdU expression patterns combined with *Nurr1 in situ* hybridization and DAPI staining (Figures 4–9). High resolution images can be accessed here: https://figshare.com/s/9e8fc7cc66be7d9e968b. We briefly describe EdU labeling in selected regions for each injection day, while a more detailed description of EdU and *Nurr1* co-labeling follows afterward. EdU injections on E12.5 (Figure 4) reveal mainly signals in the piriform cortex (Pir), intermediate endopiriform nucleus (IEn), anterior cortical amygdaloid area (ACo), basomedial amygdaloid nucleus, anterior part (BMA), medial amygdaloid nucleus (Me), medial amygdaloid nucleus, posteroventral part (MePV). Apparently, there are also some labeled cells in the incipient Sp at E12.5. Following EdU injections on E13.5 (Figure 5) we find additional EdU signals in the claustrum (CL), dorsal endopiriform nucleus (DEn), insular cortex (Ins), Sp, basomedial amygdaloid nucleus (BM), medial amygdaloid nucleus, posterodorsal part (MePD), basomedial amygdaloid nucleus, posterior part (BMP), amygdalohippocampal area, posterolateral (AHiPL), and posteromedial cortical amygdaloid area (PMCo). Following EdU injections on E14.5 (Figure 6) we find reduced EdU signal in the IEn, ACo, Me, and MePV. In addition EdU signals are visible in deep layers of secondary somatosensory cortex (S2), basolateral amygdaloid nucleus, anterior part (BLA) and the basolateral amygdaloid nucleus, posterior part (BLP). EdU injections on E15.5 (Figure 7) show reduced EdU in the DEn, deep layers of Ins, medial and posterior Pir, and the lateral part of Sp. In addition, EdU signals are visible in the superficial layers of Ins, intermediate layers of S2, secondary auditory cortex, dorsal area (AuD), and primary visual cortex (V1). Following EdU injections on E16.5 (Figure 8), EdU signals are reduced in the CL, superficial layers of Ins, intermediate layers of S2, and the dorsal part of Sp. In addition EdU signals are visible in superficial layers of AuD, V1. EdU injections on E17.5 (Figure 9) show reduced EdU signals in superficial layers of AuD but still show signals in the superficial layers of V1.

### Gradients of Neurogenesis in the Claustrum and Dorsal Endopiriform Nucleus

**FIGURE 10:**
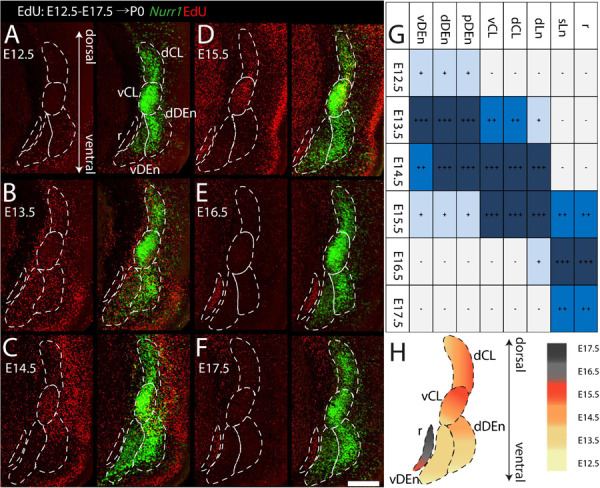
Gradients of neurogenesis in the anterior *Nurr1* labeled claustrum and its related regions. High magnification *Nurr1* labeled anterior claustrum regions, including dCL, vCL, dDEn, vDEn, with EdU labeling on E12.5 **(A)**, on E13.5 **(B)**, on E14.5 **(C)**, on E15.5 **(D)**, on E16.5 **(E)**, on E17.5 **(F)**. **(G)** Block diagram for EdU labeling signal in claustrum and its related regions from E12.5 to E17.5. White and “–” mean minimal signal, light blue and “ + ” indicate low EdU signal, stronger EdU signal is shown in darker color and more “ + ” signs. **(H)** Schematic diagram of E12.5 to E17.5 EdU labeling anterior claustrum regions. EdU signals in different regions from E12.5 to E17.5 shown as indicated by the color bar. Scale bar in panel **(A)** = 300 μm.

To study the birth dates of *Nurr1* positive neurons, we first describe EdU signals at the level of subregions, focusing first on dCL, vCL, dDEn, vDEn (Figure 10). On E12.5 (Figure 10A), we find few EdU signals in the vDEn, dDEn, vCL, and dCL. On E13.5 (Figure 10B), we find greatly increased EdU signals in the dDEn and vDEn, with some signals in the vCL and dCL. We find that the ventral part of the vCL shows more EdU signals than its dorsal part. On E14.5 (Figure 10C), vCL shows more EdU signal, and more EdU is visible in the dCL. In addition, there are more EdU signals in the dorsal part of the dDEn than in its ventral part. On E15.5 (Figure 10D), vCL is still densely labeled with EdU signals, but with more EdU signals in the dorsal part than its ventral part. The dCL still has some EdU signal, but there is less EdU in the vDEn and dDEn. A small region located medial of the DEn known as the reservoir (Bayer and Altman, 1991), paraclaustral reservoir (Maciejewska et al., 1999), or bed nucleus of the external capsule/BEC (Puelles, 2014; Puelles et al., 2016b) shows dense EdU labeling in its ventral part but does not express *Nurr1*. On E16.5 (Figure 10E) and E17.5 (Figure 10F), few EdU signals are visible in the claustrum sub-regions, but the paraclaustral reservoir/BEC is densely labeled with EdU in its dorsal part (Figures 10E,F). In summary, EdU signals first appear in the vDEn and dDEn on E12.5, first in the vCL and dCL on E13.5, first in the paraclaustral reservoir/BEC on E15.5 (Figure 10G). In Figure 10H, we summarize the ventral to dorsal gradients in birth dating. vDEn neurons are mainly born on E13.5 and dDEn neurons are mainly born on E13.5 to E14.5, then most vCL and dCL neurons are sequentially born on E14.5 to E15.5, and paraclaustral reservoir neurons are born on E15.5 to E17.5.

**FIGURE 11:**
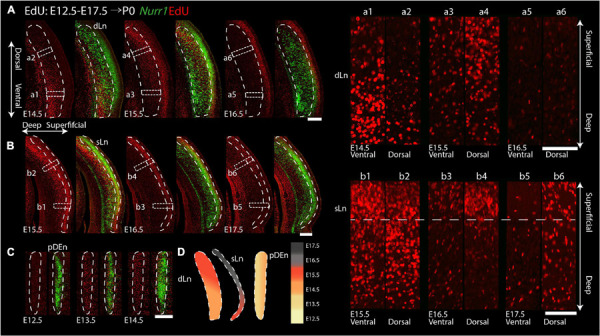
Gradients of neurogenesis in the intermediate and posterior *Nurr1* labeled claustrum related regions. High magnification *Nurr1* labeled posterior claustrum related regions with E12.5 to E17.5 EdU labeling in the dLn **(A)**, sLn **(B)**, and pDEn **(C)**. Array of crop sections of dLn (a1–a6) and sLn (b1–b6). **(D)** Schematic diagram of posterior claustrum regions. EdU signals in different regions from E12.5 to E17.5 shown as indicated by the color bar. Scale bar in panel **(A–C)** = 300 um. Scale bar in panel (a1–b6) = 100 um.

For the remaining *Nurr1* positive regions (dLn, sLn, pDEn), we study more posterior sections (Figure 11). On E14.5, we notice EdU signals mainly in the ventral parts of dLn (Figures 11Aa1,a2). On E15.5, we find EdU signals mainly in the dorsal superficial layer parts of dLn (Figures 11Aa3,a4). On E16.5, few EdU signals are in the dLn (Figures 11Aa5,a6). On E15.5, EdU signals are mainly in the ventral parts of sLn (Figures 11Bb1,b2). On E16.5 and E17.5, we observe EdU signals mainly in the dorsal parts of sLn (Figures 11Bb3–b6). On E12.5, there is little EdU signal in the pDEn. However, on E13.5 and E14.5, the pDEn is densely labeled with EdU (Figure 11C). In summary, EdU signals appear in the dLn mainly on E14.5 and E15.5, in the sLn mainly on E15.5 to E17.5, in the pDEn mainly on E13.5 and E14.5 (Figures 10G, 11D). However, we note that both dLn and sLn are intermingled with other *Nurr1* negative cortical neurons, therefore, we proceed to directly quantify *Nurr1* overlap with EdU.

**FIGURE 12:**
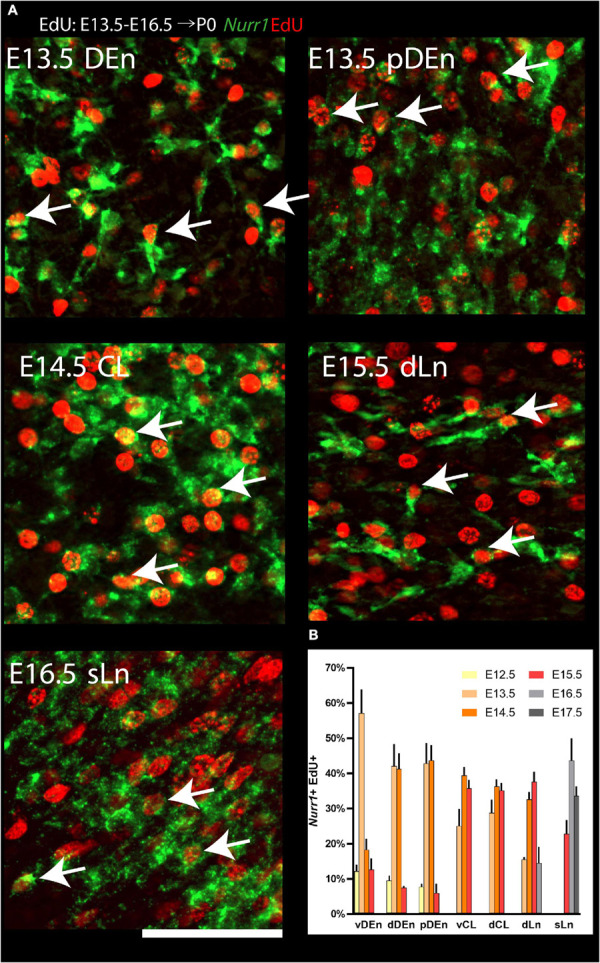
Quantification of *Nurr1* + EdU + double positive cells. **(A)** Examples of cell level overlapping of *Nurr1* and EdU in all *Nurr1* labeled claustrum regions, including DEn, pDEn, CL, dLn and sLn. **(B)** Quantification of *Nurr1* + EdU + populations born at E12.5 to E17.5 in *Nurr1* labeled regions including, dDEn, vDEn, pDEn, vCL, dCL, dLn, and sLn. Bars represent mean ± standard deviation. *N* = 3 brains for each date (3 sections from each brain). Scale bar in panel **(A)** = 100 μm.

We quantified the fraction of *Nurr1* + neurons at P0 that were born on a particular embryonic day (Figure 12), by determining how many of the *Nurr1* + neurons (green) co-localized (*Nurr1* + EdU +) with EdU + neurons (red). In the vDEn, we find the largest fraction of *Nurr1* + EdU + cells on E13.5 (57% ± 6.9). In the dDEn, most *Nurr1* + EdU + cells are born on E13.5 and E14.5 (42% ± 6.3, 41% ± 4.5). Similarly, in the pDEn, most *Nurr1* + EdU + cells appear on E13.5 and E14.5 (43% ± 5.8, 44% ± 4.4). In the vCL, most *Nurr1* + EdU + cells are born on E14.5 and E15.5 (39% ± 2.5, 36% ± 2.5). Likewise, in the dCL, we find the largest fraction of *Nurr1* + EdU + cells on E14.5 and E15.5 (36% ± 2.1, 35% ± 2.2). Also, in the dLn, we find most *Nurr1* + EdU + cells on E14.5 and E15.5 (33% ± 2.2, 37% ± 2.9). In the sLn, most *Nurr1* + EdU + cells show on E16.5 and E17.5 (44% ± 6.2, 34% ± 2.7) (Figure 12B). In summary, we find that all regions show double positive cells on more than one day. Most vDEn neurons are born by E13.5, followed by dDEn and pDEn on E13.5 to E14.5, vCL, dCL, and dLn on E14.5 to E15.5, and sLn on E15.5 to E17.5. Based on the birth dates of the sub-regions above, we find dDEn is most similar to pDEn, while vCL, dCL, and dLn are also similar. In contrast, vDEn and sLn show distinct neurogenetic patterns.

**FIGURE 13:**
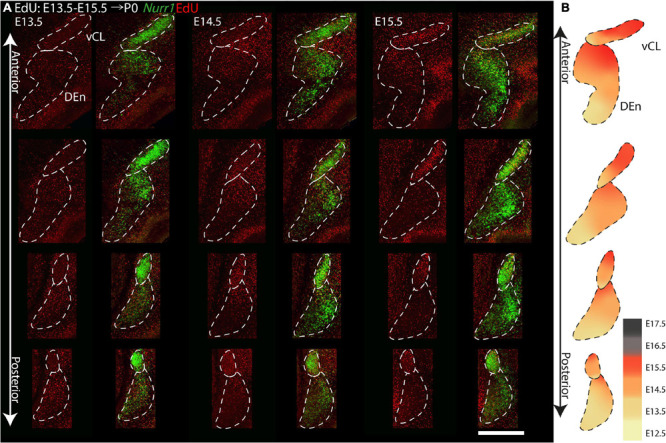
Gradients of neurogenesis in the *Nurr1* labeled CL and DEn from anterior to posterior. **(A)** High magnification *Nurr1* labeled posterior claustrum related regions, including CL and DEn with E13.5 to E15.5 EdU labeling. **(B)** Schematic diagram of the CL and DEn. EdU signals in different regions from E12.5 to E17.5 shown as indicated by the color bar. Scale bar in panel **(A)** = 0.5 mm.

To investigate anterior to posterior differences in birth dates of CL and Den, we show four comparable sections with injections between E13.5 and E15.5 (Figure 13). We focus on CL and DEn, since further subdivisions are not easy to define consistently at all anterior posterior levels. On E13.5, we find less EdU signals in vCL anteriorly, but more EdU signals in DEn posteriorly. On E14.5, we find that vCL is densely labeled with EdU except in the most anterior section. We also observe decreased EdU signals in the ventral part of DEn. On E15.5, we find decreased EdU signals in the ventral part of vCL, and also decreased EdU signals in the dorsal part of DEn (Figure 13A). In summary, EdU signals appear in the posterior DEn mainly on E13.5 and E14.5, but in the anterior DEn mainly on E13.5, E14.5 and E15.5. EdU labels the posterior CL mainly on E14.5, but the anterior CL mainly on E14.5 and E15.5 (Figure 13B).

## Discussion

To understand the cell types, structure, and function of the claustrum, and its changes in brain disorders it is critical to study the development of claustrum. Understanding the sequence of birth dates of specific types of neurons is a key aspect of neural development, but studies of claustrum birth dates have led to conflicting results (reviewed in Puelles, 2014; Bruguier et al., 2020). In addition, recent models of claustrum development and transcriptomic studies of cortical cell types indicate that neurons expressing claustrum-specific gene signatures are widely distributed within the cortex (Arimatsu, 1994; Puelles, 2014; Zeisel et al., 2015; Rosenberg et al., 2018; Yao et al., 2021), yet little is known about the development, connectivity, and function of these cortical claustrum-like cell types. This suggests several interesting questions about the development of the claustrum and other claustrum-like neurons: (i) When are these transcriptionally similar neurons generated? (ii) How do they come to be distributed so widely throughout the cerebral cortex? (iii) In which part of the developing forebrain do they originate? We first briefly summarize our findings and then in turn discuss the expression of *Nurr1* in the developing pallium, previous birth dating studies of claustrum neurons, and neurogenetic gradients in the claustrum complex.

In this study, we investigated the expression of *Nurr1* in combination with EdU labeling to map the development and birth dates of the claustrum and *Nurr1* positive neurons in the lateral cortex in brain development. The function of *Nurr1* and its role in development of dopaminergic neurons has been studied in detail (Zetterström et al., 1997; Sakurada et al., 1999; Hermanson et al., 2003; Eells et al., 2006). Interestingly, a recent study has found that in the medial pallium *Nurr1* is repressed by *Satb2* and promotes subiculum identity in the absence of *Satb2* (Zhang et al., 2020). However, so far little is known about the role of *Nurr1* expression in claustrum development. We took advantage of the early and stable expression of *Nurr1* throughout brain development to study the prenatal and postnatal ontogenetic profile of claustrum neurons. We find that *Nurr1*-labeled claustral sub-regions emerge gradually in prenatal development. We observe the first *Nurr1* staining in the prospective area of CL/DEn on E13.5 and first distinguish the ventral subdivision of the DEn by its lighter staining on E15.5. The first separation of the vDEn, dDEn and CL, and first staining of the sLn become visible on E17.5. The first clear division of dCL and vCL is apparent and first staining of the dLn on E19.5, and all sub-regions show distinct staining on E20.5 (Figure 2). We also notice the remarkable change of *Nurr1* expression in the cortex in early postnatal development. In sLn, *Nurr1* expression is reduced significantly from P2 to P20. In dLn, we observe *Nurr1* expression distantly from the subplate on P0 and closer to the subplate on P20 (Figure 3). Aside from the reduction in *Nurr1* expression in the sLn compartment, the overall expression pattern of *Nurr1* studied here remains essentially stable in postnatal development, although there is some evidence for a gradual reduction of *Nurr1* expression intensity (Crispino et al., 1998). Very little is known about the functional role *Nurr1* expression in the adult brain. In the hippocampus *Nurr1* expression levels may affect long-term memory function (Peña de Ortiz et al., 2000; Colón-Cesario et al., 2006) and Alzheimer’s disease pathology (Moon et al., 2015, 2019). It has been suggested that *Nurr1* expression may be influenced by behavioral and sensory stimuli (Biegler et al., 2021). However, supporting evidence in the mammalian brain, particularly in the claustrum, remains scarce. Even high levels of neuronal activity induced by epileptic seizures did not lead to significant changes of *Nurr1* expression in the early postnatal rat brain (Crispino et al., 1998). In later postnatal stages and in adult animals seizure induced changes in *Nurr1* expression levels were mainly detected in the hippocampus (Xing et al., 1997; Crispino et al., 1998; Kummari et al., 2021). Clearly, further studies are needed to understand the relation of neuronal activity and *Nurr1* expression in the mammalian forebrain. By using EdU labeling, we detect dividing neurons in combination with *Nurr1* FISH (Figures 4–9). We find that most *Nurr1* positive neurons in the vDEn are born on E13.5, in the dDEn and pDEn mainly on E13.5 and E14.5, in the vCL and dCL mainly on E14.5 and E15.5, in the dLn mainly on E14.5 and E15.5, and in the sLn mainly on E15.5, E16.5 and E17.5 (Figures 10, 12). Finally, we identify ventral to dorsal and posterior to anterior gradients of birth dates in subregions including the vCL and DEn (Figures 10, 11, 13).

### *Nurr1* Expression in the Developing Forebrain

**FIGURE 14:**
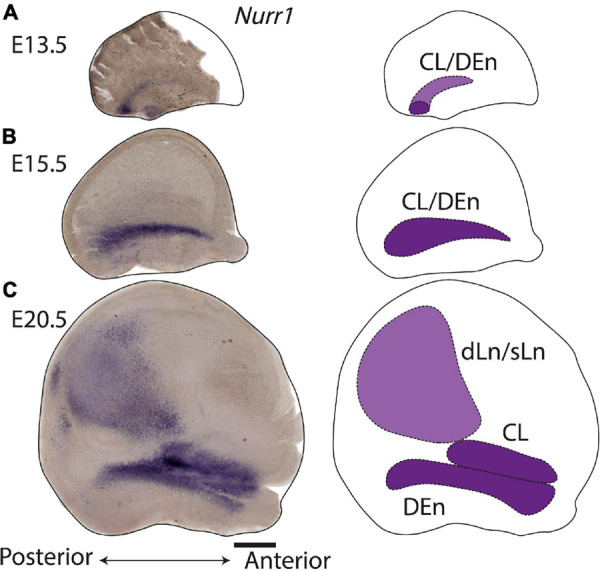
Overview of claustrum development. Flattened cortex and schematic diagram of *Nurr1* expression by *in situ* in E13.5 **(A)**, E15.5 **(B)**, and E20.5 **(C)**. One hemisphere was flattened and sectioned into 100 μm sections. Multiple sections were overlayed in panels **(B,C)**. Scale bar in panel **(A)** = 1 mm.

In the claustrum, *Nurr1* expression has been reported in the prospective primordium of claustrum in the superficial part of lateral pallium as early as E12.5 in mice (Puelles, 2014). Rat embryonic brain development is thought to attain a comparable state about one to two days later than in mice (Schneider and Norton, 1979; Clancy et al., 2001). Consequently, we find the first *Nurr1* staining in the lateral forebrain on E13.5. However, we note that *Nurr1* staining on E13.5 in the rat shows some distance to the surface of pallium, with its ventral part relatively closer to the pallium surface than its dorsal parts (Figure 2Aa). Interestingly, while the prospective claustrum neurons remain relatively weakly stained at this time point, we find that there is a more strongly stained posterior-ventral *Nurr1* positive region. By following a series of coronal sections (Figure 2A), or on sections cut parallel to the surface (Figure 14A), we find that both *Nurr1* positive regions appear to be continuous.

While Puelles (2014) proposed that *Nurr1* positive claustrum neurons are generated in the lateral pallium, a recent study by Moreau et al. (2021) finds that in the mouse on E12.5 *Nurr1* positive neurons are located in the ventral pallium. One possibility is that these cells are unrelated to claustrum neurons and reduce their *Nurr1* expression over time. However, if they are related to claustrum neurons, their appearance in different sectors of the pallium remains unexplained (Moreau et al., 2021). Cell lineage analysis supports the former view, suggesting that claustrum and endopiriform neurons are indeed generated in the lateral pallium (Garcia-Moreno et al., 2018; Rueda-Alaña et al., 2018; Bruguier et al., 2020). By E15.5, the majority of *Nurr1* staining has congregated in a lateral stripe in the prospective CL/DEn region (Figures 2B, 14B). Little staining remains visible in the previously strongly labeled posterior-ventral parts of the pallium, indicating either loss of *Nurr1* expression, or depletion of *Nurr1* positive neurons in that region (Figures 2B, 14B). Consistent with the results of Puelles (2014), we find that over the next days of embryonic development the formerly uniform elongated *Nurr1* positive region differentiates into multiple subdivisions (Figures 2B–D). Among those, the earliest visible subdivision is based on the light and dark staining of *Nurr1* in the DEn, dividing it into ventral and dorsal parts (vDEn and dDEn, Figure 2Bb2). This segmentation of the DEn can also be observed in adult animals by using additional markers including *Lxn*, *Ntng2, and Oprk1*, which are enriched in dDEn, and *Cpne4, Pcp4, and Npsr1*, which are enriched in vDEn (Arimatsu, 1994; Naumann et al., in preparation). The molecular characterization of cell types in the endopiriform nucleus remains largely unexplored (Smith et al., 2019). However, identifying mouse lines with specific expression in subsets of DEn neurons (Riedemann et al., 2019) will enable elucidating their development, connectivity, and function.

In more dorsal parts of cortex, *Nurr1* positive sLn neurons first appear faintly on E17.5 (Figure 2Cc3), while dLn neurons first become visible somewhat later, on E19.5 (Figure 2Dd2), comparable to descriptions of dLn in the mouse (Puelles, 2014; Ratié et al., 2020). By E20.5, both sLn and dLn neurons are found in a broad region throughout the posterior dorsal cortex (Figures 2E, 14C). Interestingly, sLn and dLn neurons show a sharply divergent postnatal development (Figure 3). In the sLn region, *Nurr1* expression is strongly reduced over the first 10 days of postnatal development and essentially absent by P20. In contrast, dLn neurons become more strongly stained and appear to settle in deeper layers. This observation may be explained by a coordinated pattern of up - and down-regulation of *Nurr1* expression involving multiple neuronal populations at different distances from the subplate during early postnatal development. An alternative explanation for such coordinated changes in Nurr1 expression was suggested by Li et al. (2011). Using antibodies against Nurr1, they observed similar changes in staining patterns in the cortex and embryonic spinal cord, interpreting the latter as evidence for neuronal migration. Interestingly, *Nurr1* may have a role in neuronal migration during the development of midbrain neurons (Wallén et al., 1999), however, no evidence for such a function is available for *Nurr1* in forebrain neurons. In addition, we note that, while the generation of the laminar pattern of cortical neurons is essentially complete before birth (Figures 1C,D), cortical thickness around dLn neurons expands 3-fold (Figure 3C), similar to other cortical regions (Bandeira et al., 2009; Ray and Brecht, 2016). This expansion of cortical thickness may confound the analysis of laminar position of dLn neurons. As pointed out previously, direct evidence for any of these migratory patterns is lacking (Bruguier et al., 2020), and further cell lineage tracing studies are necessary to understand the changing patterns of *Nurr1* expression during development. Such studies should also take into account the complex developmental patterns of other cortical neurons (Saito et al., 2019).

### Methods for Birthdating

As discussed by Puelles (2014), differences in pin-pointing the birth dates of claustrum neurons may stem from methodological issues. Bayer and Altman (1991) and earlier studies used radioactive [H^3^]Thymidine given at a single or multiple time points. In [H^3^]Thymidine studies, cell types are mainly inferred by their anatomical location. Arimatsu (1994) and Arimatsu et al. (2003) used antibodies against Latexin or Nurr1 in combination with the thymidine analog 5-bromo-2′-deoxyuridine (BrdU) to uncover the birthdates of claustrum and Nurr1/Lxn positive neurons in the lateral cortex. However, BrdU detection requires aggressive denaturation which may impact co-labeling with antibodies or *in situ* hybridization (Miller and Nowakowski, 1988; Chehrehasa et al., 2009). Administration of BrdU may also interfere with neuroblast proliferation and promote apoptotic cell death (Rodríguez-Vázquez and Martí, 2021). Therefore, we decided to use the more recently developed thymidine analog EdU, which does not require DNA denaturation and has been shown to be a specific and reproducible labeling tool to study cell proliferation in the adult central nervous system (Zeng et al., 2010). We also considered FlashTag labeling which allows for birth dating in a more precisely defined time window (Govindan et al., 2018). However, FlashTag labeling, so far, appears restricted to investigating dorsal cortex progenitors (Yoshinaga et al., 2021). One drawback of EdU is its even higher toxicity than BrdU at comparable dosing regimes (Ross et al., 2011; Andersen et al., 2013). However, since EdU is significantly more easily detected, it was possible to administer EdU at a fraction of the dose of BrdU in previous studies (Figure 1B; Arimatsu et al., 2003). We found that even at the lowest injection dose tested (10 mg/kg), EdU remained readily detectable using our staining protocol. The overall EdU labeling pattern in general agrees with previous birth dating studies. For example, in the cortex there is a clear inside-out EdU labeling pattern and lateral parts of the cortex show precocious development compared with dorsal regions (Figures 1C,D; Hicks and D’Amato, 1968; Smart and Smart, 1977; Saito et al., 2019). Further studies combining, for example, BrdU and EdU as shown by Harris et al. (2018), but using injections on different days, will enable direct comparisons of cells born at different time points. While we focus on EdU labeling in the claustrum and related cell types, our data may be of use to researchers interested in the birth dating of other brain regions, therefore we provided high resolution versions of Figures 4–9 online.^1^

### Birth Dating and Neurogenetic Gradients of *Nurr1* Positive Neurons

Previous birth dating studies of claustrum and DEn neurons have led to conflicting results (see Puelles, 2014, 2017; Bruguier et al., 2020 for reviews). Some earlier studies had suggested that rat claustrum neurons are born before E12.5 (Bisconte and Marty, 1975; Valverde and Santacana, 1994), however, our data indicate that the majority of all claustrum, DEn, and related *Nurr1* positive neurons are born after E12.5 (Figure 10). We think that using reference marker, such as *Nurr1*, greatly expedites the assignment of birth dates to defined neuronal populations. Presumably, the lack of such a reference marker explains the major differences in the interpretation of the data in Bisconte and Marty (1975) and Valverde and Santacana (1994). Further differences may stem from the use of thin sections in [H^3^]thymidine studies (typically 5 μm), whereas we use relatively thick sections (50 μm), and differences in defining embryonic days. Our claustrum birth dating results agree to a larger extent with the findings of Bayer and Altman (1991); Arimatsu (1994), and Arimatsu et al. (2003). However, it should be noted that Bayer and Altman (1991) define the first day of embryonic development as E1, while Arimatsu (1994) and Arimatsu et al. (2003) define it as E0, whereas we define it as E0.5. For example, Arimatsu (1994) observe that the majority of DEn neurons are born on E13, we find that they are mainly born on E13.5 to E14.5, while Bayer and Altman (1991) show DEn birth dates from E14 to E15. In addition, we further subdivided DEn into three regions. vDEn has lower *Nurr1* expression levels and shows the earliest birth dates (mainly on E13.5), whereas dDEn and pDEn have higher *Nurr1* expression and later birth dates (on E13.5 and E14.5). Medial to the vDEn, we observed a group of cells called reservoir or paraclaustral reservoir (Bayer and Altman, 1991; Maciejewska et al., 1999) or more recently, bed nucleus of the external capsule (BEC) (Puelles, 2014; Puelles et al., 2016b). Interestingly, these cells show distinctly later birth dates (E15.5-E17.5) than the neighboring DEn (Figures 7–10). These *Nurr1* negative neurons are either thought to migrate into olfactory cortex regions within the first few days after birth (Maciejewska et al., 1999) or constitute a more stable nucleus (Puelles, 2014; Puelles et al., 2016b). In both cases, they are unlikely to be related to claustrum neurons. When considering the adjacent cortical regions, we find that the olfactory cortex cells are produced from E13.5 to E15.5 and insular cortex cells are generated mainly on E14.5 to E15.5, indicating that DEn and claustrum develop jointly with their surrounding cortical regions (Puelles et al., 2016a). In contrast, the paraclaustral reservoir/BEC cells are born significantly later than the adjacent olfactory cortex neurons. Again, when we divide the claustrum into the vCL and dCL regions, they show largely similar birth dating patterns. Interestingly, the dLn *Nurr1* positive neurons are generated largely on the same days as claustrum neurons whereas sLn neurons are generated significantly later (Figures 11, 12). Together with the strong reduction in *Nurr1* expression during early postnatal development, this indicates that sLn neurons constitute a distinct neuronal type, whereas dLn neurons are more closely related to other claustrum neurons, in agreement with their claustrum-like gene expression profile (Bruguier et al., 2020; Peng et al., 2021).

Bayer and Altman (1991) describe a ventral to dorsal neurogenetic gradient in the DEn and a posterior to anterior neurogenetic gradient in the claustrum. In general, our data also support the notion of neurogenetic gradients in CL and DEn, but we find that both types of neurogenetic gradients are present in DEn and claustrum (Figures 10, 13). Thus, *Nurr1* positive neurons are born earlier in ventral and posterior regions and later in dorsal and anterior regions within the major claustrum and DEn subregions. In contrast to Arimatsu (1994) and Arimatsu et al. (2003), we also identify ventral to dorsal neurogenetic gradients in lateral cortical *Nurr1* positive dLn and sLn neurons (Figure 11). Overall, the prenatal development of *Nurr1* expression follows the birth dating pattern, although it remains to be determined how long after neuronal production *Nurr1* expression is induced. For example, claustrum and DEn neurons can be differentiated by their birth dates between E13.5 and E15.5, but based on *Nurr1* staining alone, these subdivision are only recognizable after E17.5. Thus, experiments combining EdU injections with *Nurr1* staining during embryonic development could provide clues about the relative timing of neuron production and induction of *Nurr1* expression. More detailed fate-mapping studies are required to determine the origin and trajectory of claustrum and *Nurr1* positive neurons in the lateral cortex.

## Conclusion

Using *Nurr1* we comprehensively studied the development of the claustrum and the *Nurr1* positive neurons in the lateral cortex in the prenatal and early postnatal rat brain. We provide a detailed segmentation of claustral sub-regions and identify transient changes of *Nurr1* expression in the cortex. By combining EdU labeling and ISH, we investigated the birth dates of sub-regions of the claustrum and related *Nurr1* positive neurons in the lateral cortex. We describe the birth dating patterns of all sub-regions, which helps to understand their relationships based on their neurogenetic pattern. Also, we show that a ventral to dorsal and posterior to anterior neurogenetic gradient is broadly conserved within claustrum and DEn sub-regions. In summary, our data charts the embryonic and early postnatal patterns of claustrum development, and contributes toward explorations of claustrum function and dysfunction during early life.

## Data Availability Statement

The original contributions presented in the study are included in the article/supplementary material, further inquiries can be directed to the corresponding author.

## Ethics Statement

The animal study was reviewed and approved by Animal Care and Use Committees at the Shenzhen Institute of Advanced Technology (SIAT), Chinese Academy of Sciences (CAS).

## Author Contributions

CF, RN, and HW: experimental design and critical revision of the manuscript for important intellectual content. CF and RN: data acquisition and analysis and drafting of the manuscript. RN and HW: obtain funding, administrative, technical, and material support, and study supervision. All authors contributed to the article and approved the submitted version.

## Conflict of Interest

The authors declare that the research was conducted in the absence of any commercial or financial relationships that could be construed as a potential conflict of interest.

## Publisher’s Note

All claims expressed in this article are solely those of the authors and do not necessarily represent those of their affiliated organizations, or those of the publisher, the editors and the reviewers. Any product that may be evaluated in this article, or claim that may be made by its manufacturer, is not guaranteed or endorsed by the publisher.

## Footnotes

1
https://figshare.com/s/9e8fc7cc66be7d9e968b

## Abbreviations

ACo: anterior cortical amygdaloid area
AHiPL: amygdalohippocampal area, posterolateral
AuD: secondary auditory cortex, dorsal area
BEC: bed nucleus of the external capsule
BMA: basomedial amygdaloid nucleus, anterior part
BLA: basolateral amygdaloid nucleus, anterior part
BLP: basolateral amygdaloid nucleus, posterior part
BM: basomedial amygdaloid nucleus
BMP: basomedial amygdaloid nucleus, posterior part
CL: claustrum
dCL: dorsal claustrum
DEn: dorsal endopiriform
dDEn: dorsal DEn
dLn: deep layer neurons
EP: endopiriform nucleus
IEn: intermediate endopiriform nucleus
Ins: insular cortex
Me: medial amygdaloid nucleus
MePD: medial amygdaloid nucleus, posterodorsal part
MePV: medial amygdaloid nucleus, posteroventral part
PMCo: and posteromedial cortical amygdaloid area
S2: secondary somatosensory cortex
pDEn: posterior DEn
Pir: piriform cortex
r: paraclaustral reservoir
sLn: superficial layer neurons
Sp: subplate
V1: primary visual cortex
vCL: ventral claustrum
vDEn: ventral DEn.
